# Historia de las epidemias de sarampión en Colombia: un conteo incompleto

**DOI:** 10.7705/biomedica.7650

**Published:** 2025-09-22

**Authors:** Claudia Amaya-Castellanos, Francisco Ortega, Álvaro J. Idrovo

**Affiliations:** 1 Universitat Rovira i Virgili, Tarragona, España Universitat Rovira i Virgilu Universitat Rovira i Virgili Tarragona Spain; 2 Departamento de Salud Pública, Escuela de Medicina, Universidad Industrial de Santander, Bucaramanga, Colombia Universidad Industrial de Santander Departamento de Salud Pública Universidad Industrial de Santander Bucaramanga Colombia; 3 Instituciò Catalana de Recerca i Estudis Avancats (ICREA), Barcelona, España Instituciò Catalana de Recerca i Estudis Avancats Barcelona España

**Keywords:** sarampión/epidemiología, historia, epidemias, salud de poblaciones indígenas, Colombia, measles/epidemiology, history, epidemics, health of indigenous peoples, Colombia

## Abstract

Las epidemias de sarampión emergieron en el territorio colombiano con la llegada de los europeos durante la Conquista y la Colonia, ocasionando la muerte de miles de indígenas. Poco se sabe de epidemias posteriores.

Este artículo resume la historia de 36 epidemias de sarampión ocurridas en Colombia y se concentra en la primera epidemia entre los indígenas hitnü (1964) para mejorar la comprensión de algunos hechos históricos. Pese al subregistro, se identificaron grandes epidemias ocurridas en territorios indígenas (siglos XVI a XVIII), Salamina (1885) y Bogotá (1905-1906). Fue evidente que la falta de inmunidad tuvo mayor impacto durante los periodos de la Conquista y la Colonia.

Los factores determinantes socioculturales siempre condicionaron el acaecimiento de las epidemias, pero sus efectos son mayores en periodos posteriores a la disponibilidad de vacunas. La violencia directa y la estructural han sido los factores determinantes más importantes de las epidemias de sarampión en el país. Actualmente, hay una reemergencia mundial que amenaza a Colombia.

## Abstract (en)

Measles epidemics emerged in Colombia with the arrival of Europeans during the Conquest and Colonial periods, causing the death of thousands of indigenous people. Little is known about subsequent epidemics.

Here, we summarize the history of 36 measles epidemics in Colombia, focusing on the first epidemic among the Hitnü indigenous people (1964) to improve the understanding of some historical facts. Despite underreporting, major epidemics were identified in indigenous territories (16^th^ to 18^th^ centuries) in Salamina (1885) and Bogotá (1905-1906). It was evident that the lack of immunity had a greater impact during the Conquest and Colonial periods. Sociocultural determinants have always influenced the occurrence of epidemics, but their effects are greater after vaccines become available. Direct and structural violence have been the most important determinants of measles epidemics in the country. Currently, a global reemergence threatens Colombia.

La población de los pueblos aborígenes americanos se disminuyó durante los periodos de la Conquista y la Colonia, en gran medida, debido a la llegada de microorganismos frente a los que no tenían inmunidad ^1^. Sin embargo, durante más de cinco siglos varios grupos indígenas permanecieron en relativo aislamiento del resto del mundo, principalmente en regiones selváticas de Suramérica, razón por la cual se pospuso el contacto de estas comunidades con individuos portadores de microorganismos potencialmente catastróficos ^2^. Por esta razón, no tuvieron exposición al virus del sarampión, el cual causó gran morbilidad y mortalidad a nivel global antes de aparecer la vacunación.

En este contexto, este trabajo presenta brevemente la historia de las epidemias de sarampión ocurridas en el territorio colombiano. El estudio de varias epidemias en diferentes momentos históricos permite explorar su grado de similitud o diferencia en términos de susceptibilidad, vulnerabilidad y resiliencia ^3^. Un análisis de este tipo permite determinar si los patrones de estos tres elementos se comportan de manera similar a lo largo del tiempo o si no lo hacen. Se parte de los supuestos de que: 1) los "primeros encuentros" entre un microorganismo emergente y los aborígenes sin inmunidad son desastrosos, y 2) cuando ya existen las vacunas, la afectación será heterogénea entre los grupos humanos, dependiendo de sus grados de vulnerabilidad y susceptibilidad y el acceso a la vacuna, principal modificador de la resiliencia. De esta manera, las epidemias corresponden a "experimentos naturales" de los procesos sociohistóricos que acrecientan las desigualdades en la salud poblacional ^4^, y permiten evidenciar las inequidades de la sociedad.

Para cumplir con el objetivo, se revisaron las fuentes históricas escritas disponibles, lo que constituye un primer intento de revelar la historiografía de las epidemias de sarampión en Colombia. Además, se presentan los hechos ocurridos durante la primera epidemia de sarampión (1964) entre los indígenas hitnü, un grupo de tradición nómada que habita en la región del Arauca colombiano ^5^. Con la comprensión de esta epidemia se busca, desde el presente, mejorar el entendimiento de las epidemias del pasado; en especial, aquellas que ocurrieron entre indígenas, antes de la introducción de las vacunas en poblaciones vulnerables. Entendemos, como bien dice el historiador de la medicina Diego Armus, que la historia no trae enseñanzas ni guías para no equivocarse en el futuro; solo muestra directrices generales de la complejidad del pasado. Más bien, los hechos presentes pueden ayudar a entender el pasado buscando las similitudes en los acontecimientos ^6^.

El enfoque de este trabajo es el de la epidemiología histórica, es decir, un punto de encuentro entre la historia de la medicina y la epidemiología. Para James Webb, la epidemiología histórica es el estudio del impacto que tienen todos aquellos esfuerzos que se realizan para controlar las enfermedades a lo largo del tiempo, y las formas en que las intervenciones han transformado los patrones de las enfermedades, e influido en su transmisión. Integra procesos ecológicos, sociales, económicos y políticos, con los procesos patológicos, las respuestas humanas y los efectos de las intervenciones sanitarias globales ^7^.

Este enfoque surgió de la geografía médica, cuyo pionero, Erwin H. Ackerknecht, empezó a estudiar la malaria hacia 1940 en el valle del río Misisipi con conocimientos sociales, económicos y ambientales, para comprender los cambios temporales y espaciales en la presentación de la enfermedad ^8^. Dos décadas después, Philip Curtin realizó importantes estudios sobre la historia de las enfermedades infecciosas en África, incluso, estimando tasas de morbilidad, mortalidad y supervivencia ^9^. Estos trabajos seminales fueron seguidos por otros estudios de epidemiología histórica sobre diversas enfermedades en variadas regiones del mundo, sin que sea una aproximación usual de la historia de la medicina.

En Colombia, hay algunos ejemplos de estudios de epidemiología histórica, entre los cuales sobresalen los múltiples trabajos de Hugo Armando Sotomayor, como *Historia y geografía de algunas enfermedades en Colombia*^10^. Se adicionan estudios de varios autores, con variable influjo de la historia y la epidemiología sobre enfermedades como la tuberculosis en Bogotá ^11^, las fiebres del Magdalena ^12^^,^^13^, la lepra en Boyacá ^14^, las epidemias de viruela de 1782 y 1802^15^, la pandemia de gripe de 1918-1919 ^16^, y el hidrargirismo en la mina de mercurio de Aranzazu (Caldas) ^17^, entre otros.

## El sarampión y su origen

El sarampión, llamado así según algunas versiones por las palabras latinas *misellus* o *misella,* diminutivo de *miser,* cuyo significado es miseria ^18^, fue una enfermedad introducida en América por los europeos durante el periodo de la Conquista ^19^, y de rápida dispersión cuando las condiciones fueron propicias dada su gran contagiosidad. Incluso, con modelos matemáticos recientes, se informa que el número reproductivo básico *(R*_*0*_ *),* o el promedio de casos incidentes asociados con un caso, puede llegar a 15 ^20^, aunque en ocasiones se han descrito números mayores.

El agente causal es un virus del género *Morbillivirus,* similar a virus encontrados en mamíferos marinos como delfines y marsopas, perros y rumiantes como las vacas. El más parecido es el virus de la peste bovina (*Rinderpest virus*)*,* lo que sugiere un origen común cuando humanos y vacas empezaron a compartir el hábitat en el Viejo Mundo ^21^. Los resultados de varios análisis del reloj molecular, que permiten estimar la edad de los ancestros en la historia evolutiva mediante patrones filogenéticos, sugieren que la divergencia del virus del sarampión y el virus de la peste bovina ocurrió en diversos periodos, dependiendo de las muestras analizadas ^22^^-^^26^; las más confiables son las de los análisis de Wertheim y Kosakovsky, que indican que fue en el año 899 (IC 95 %: 597 a 1144) ^25^. Sin embargo, Düx *et al.* señalan que la divergencia pudo ocurrir en el neolítico, hacia los años 2821 a. n. e. (IC 95 %: 4177 a 1665 a. n. e.) ^26^. Si bien estas fechas permiten identificar las primeras endemias, no es fácil rastrear desde cuándo empezaron a presentarse epidemias de grandes proporciones, debido a que el sarampión pudo haberse confundido con la viruela y la rubeola ^27^^,^^28^. La primera distinción clínica del sarampión y la viruela se atribuye al médico persa Al-Razi, quien, en el siglo VIII, propuso el aislamiento de los enfermos como forma de evitar las epidemias ^29^.

## Epidemias de sarampión

Si bien la enfermedad pudo mantener un comportamiento endémico en varias regiones después de aparecer, solo hasta el Medioevo empezaron a registrarse epidemias de sarampión en el sur y en el oriente asiáticos (incluyendo India y China), y en varios países de Europa. Entre todas, sobresale la epidemia de Inglaterra y Escocia, ocurrida entre 1670 y 1674, cuando Thomas Sydenham aprovechó para describir la enfermedad, lo cual tuvo gran impacto en la medicina practicada en los territorios con influjo europeo ^29^. Durante los siglos XIII a XX, el sarampión fue endémico en varias regiones europeas, afectando principalmente a menores de edad en condiciones de pobreza y hacinamiento ^28^.

De acuerdo con diversos historiadores como Noble David Cook, la llegada del sarampión a América fue posterior a la gripe y la viruela, y tuvo diversas expresiones: primero, como pandemia y, luego, como epidemias en diversas regiones y poblaciones del continente. Para este investigador, la primera pandemia de sarampión, ocurrida entre 1532 y 1533, afectó poblaciones de Centroamérica y de los Andes suramericanos, y causó la muerte de 25 a 30 % de la población indígena ^1^. Para el médico historiador Francisco Guerra, el sarampión llegó antes; en su revisión, indica que hubo epidemias en Santo Domingo (1496 y 1502), Puerto Rico (1508), Guatemala (1523), Panamá (1523), Cuba (1529) y México (1531) ^30^.

En el territorio de la actual Colombia, ocurrieron varias epidemias de sarampión ^30^^-^^52^^)^ (cuadro 1). La primera se presentó en los años previos a 1571, junto a otras enfermedades que habían afectado a los indígenas de la Nueva Granada ^30^. Quizá uno de los lugares a los que se hace referencia es Tunja, donde ocurrió una epidemia en 1559, que ocasionó gran número de muertos entre los indígenas ^31^. Este hecho quedó reportado por Venero de Leyva ^32^, y resulta coherente que el sarampión estuviese en los países cercanos, por la descripción de epidemias en Centroamérica, el Caribe y Perú en 1558 ^30^, desde donde pudieron haber llegado algunos individuos infectados. Es muy llamativo que las epidemias del siglo XVI se describieron fundamentalmente en las zonas de montaña, no en las regiones de ingreso al territorio colombiano, pero sí en una de las regiones donde más indígenas vivían, lo que facilitó la propagación de la infección y ocasionó un desastroso declive demográfico.

**Cuadro 1:** Principales epidemias de sarampión en Colombia a lo largo de la historia

Años	Lugar	Descripción	Referencia
1559	Tunja	Gran número de indígenas muertos	Francis, 2002 ^31^
1571	Nueva Granada	En los últimos 15 años, los indios habían sufrido […] sarampión.	Guerra, 1999 ^30^
1572	Tunja	Disminución del 57 % de la población entre 1572 y 1596 por la viruela y el sarampión	Guerra, 1990 ^30^
1616	Remedios y Zaragoza (Antioquia)	Las muertes de los indios llevaron al cierre de las minas de oro por falta de mano de obra.	Guerra, 1990 ^30^
1617-1618	Provincia de Tunja	Epidemia posterior a una hambruna por una plaga de langostas que consumió los cultivos de maíz. De allí se extendió por gran parte del país.	Francis, 2002 ^31^
1617-1618	Santa Fe de Bogotá	“Mató a más de un quinto de los indios”, sin afectar a los españoles. Previamente hubo plaga de langostas que acabó con las cosechas.	Villamarín y Villamarín, 2000 ^32^
1618	Santa Fe de Bogotá	Junto con la epidemia de 1621 ocasionaron la muerte del 20 % de los indios tributarios; los españoles fueron poco afectados.	Lucena Giraldo, 1965 ^33^
1619	Remedios y Zaragoza (Antioquia)	Epidemia que ocasionó gran mortandad	Guerra, 1999 ^30^
1619	Nueva Granada	Antes de la epidemia ocurrió una plaga de saltamontes, lo que llevó a la pérdida de las cosechas y la escasez de alimentos. En solo Fontibón, hubo 700 indios enfermos y muchos fallecidos por falta de alimentos y atención sanitaria.	Guerra, 1990 ^30^
1621	Santa Fe de Bogotá	Junto con la epidemia de 1618 ocasionaron la muerte del 20 % de los indios tributarios; los españoles fueron poco afectados.	Lucena Giraldo, 1965 ^33^
1659	Santa Fe de Bogotá y Chía	Se indica gran frecuencia entre los indígenas.	Villamarín y Villamarín, 2000 ^32^
1692	Santa Fe de Bogotá	Junto con la viruela (1693) disminuyó el 30 % de los indios tributarios; el sarampión afectó españoles (incluso 5 jesuitas) e indígenas.	Guerra, 1999 ^30^
1692-1693	Cartagena	Las epidemias de sarampión y viruela causaron numerosas bajas en la población.	Ghisays Ganem, 2014 ^34^
1729	Santa Fe de Bogotá	Gran afectación entre los indígenas, y poca entre los españoles	Villamarín y Villamarín, 2000 ^32^
1749	Curso alto del río Negro, en la frontera de Colombia, Venezuela y Brasil	Epidemia muy extensa entre el pueblo desana, llamada “el gran sarampión”, sin claro impacto en población colombiana	Buchillet, 2013 ^35^
1749	Amazonía	Epidemias en las misiones católicas del Amazonas, Napo y Ucayale	De Castellvi, 1944 ^36^
1756			
1762			
1763	Curso alto del río Negro, en la frontera de Colombia, Venezuela y Brasil	Epidemia asociada con la llegada de militares portugueses, que afectó a indígenas obligados a trabajar en plantaciones coloniales.	Buchillet, 2013 ^35^
1795-1796	Medellín	Epidemia en la que murieron “muchos niños y gentes mayores”	Gómez *et al*., 2021 ^37^
1820	Santa Fe de Bogotá	“Fuerte epidemia” de la que se ignora el número de víctimas	Ibáñez, 1968 ^38^
1869	Titiribí, Antioquia	Epidemia bastante grande de la que no hay muchos datos	Gallo, 2010 ^39^
1873	Santa Fe de Bogotá	La epidemia causó “numerosas víctimas”.	Ibáñez, 1968 ^38^
1885	Salamina, Caldas	Gran epidemia con 1.800 individuos infectados y 87 fallecidos.	Londoño, 1890 ^40^
1885-1890	Pereira, Risaralda	Epidemia de gran magnitud, sin más datos	Londoño, 1890 ^40^
1890	Antioquia	Epidemias en varios municipios; hubo epidemia de tos ferina antes.	Uribe Ángel, 1890 ^41^
1897	Varias regiones	Afectó Antioquia, Bogotá, Boyacá, Santander, Tolima y la costa Atlántica. Solo en Bogotá hubo 620 fallecidos, principalmente pobres.	Osorio et al, 1897 ^42^
1905-1906	Bogotá	Epidemia que ocasionó 326 muertes, principalmente de los barrios pobres de la ciudad	Herrera, 1906 ^43^^-^^45^
1922	Titiribí, Antioquia	El médico oficial informó que la enfermedad estaba “haciendo su agosto”.	Gallo, 2010 ^39^
1923	Putumayo	Gran mortalidad entre los indígenas siona	Mongua Calderón y Langdon, 2020 ^46^
1932-1934	Amazonía	La epidemia diezmó al pueblo yagua; asociado con la presencia de tropas peruanas.	Chaumeil, 2022 ^47^
1933	Bogotá	La mortalidad por sarampión llegó a ser casi la cuarta parte de la mortalidad total.	Velasco Cabrera, 1938 ^48^
1960-1964	Catatumbo (frontera colombo-venezolana)	Epidemia que ocasionó la muerte del 70 % del pueblo barí, principalmente en territorio colombiano	Lizarralde y Lizarralde, 2016 ^49^ Cárdenas Arroyo, 2021 ^50^
1964	Arauca	Un tercio del pueblo hitnü falleció por la epidemia.	Amaya Castellanos *et al*, 2025 ^5^
1993	Colombia	Brotes en todo el país con 5.000 casos y 48 muertes	Fraser, 2018 ^51^
2019-2020	Colombia	El Instituto Nacional de Salud reporta 24 brotes asociados principalmente con migrantes provenientes de Venezuela.	Prieto Alvarado *et al*., 2023 ^52^

En las regiones mineras de Remedios y Zaragoza (Antioquia), hubo una epidemia en 1616; las muertes de indígenas fueron tantas, que debieron suspender la extracción de oro por falta de mano de obra ^30^. Durante 1617 y 1618, hubo una epidemia en la Provincia de Tunja, posterior a una hambruna provocada por la pérdida de cultivos por una plaga de langostas ^32^. En 1618 y 1621, hubo epidemias de sarampión que ocasionaron la muerte de una quinta parte de los indios tributarios, según carta enviada al rey por don Juan de Borja, presidente de la Audiencia de Santa Fe de Bogotá ^33^. En 1619, una nueva epidemia de sarampión ocasionó una gran mortandad en la región de Remedios y Zaragoza, en Antioquia ^30^. Por los lugares afectados, es evidente que las epidemias de sarampión ocurrieron en regiones donde había mayor conglomeración y movimiento de individuos, ya fuese por ser los lugares de asentamiento prehispánico, o por la llegada europea a zonas de importancia económica para la época. De acuerdo con el médico Miguel Ángel Ghisays Ganem, en 1692 y 1693: «[...] una epidemia de viruelas y sarampión causa numerosas bajas en la población [...]» de Cartagena ^34^.

En 1749, ocurrió una epidemia entre los indígenas desana, de la zona alta del río Negro en Brasil, cerca de las fronteras actuales con Colombia y Venezuela. Este pueblo vivió una epidemia de sarampión de gran magnitud y, por ello, denominada "el gran sarampión". Después de 1763, se presentó otra con la llegada de militares portugueses que obligaban a los indígenas a trabajar en plantaciones coloniales ^35^. En la misma región, según fray Marcelino de Castellvi, hubo epidemias de sarampión en 1749, 1756 y 1762, que afectaron a los indígenas de las misiones del Amazonas, Napo y Ucayale ^36^. Durante 1795 y 1796, hubo una epidemia de sarampión en Medellín que ocasionó gran mortandad entre menores de edad y en adultos mayores ^37^. Pedro María Ibáñez, en sus *Memorias para la Historia de la medicina en Santa Fe,* describió una "fuerte epidemia" de sarampión, de la cual no hay registro numérico de los afectados. El mismo autor refiere la publicación, en 1837, al parecer muy difundida, de la *Receta para curar el sarampión* escrita por José Félix Merizalde ^38^. También, se sabe de una epidemia de sarampión en 1873 ocurrida en Santa Fe de Bogotá, aunque sin claridad del número de afectados; en ese mismo año, se publicó la *Instrucción popular para la curación del sarampión* de Nicolás Osorio y Gabriel Castañeda ^38^.

Una gran epidemia de sarampión proveniente de Perú y Ecuador llegó a finales del siglo XVII a Colombia, después de muchas muertes en ciudades como Lima y Quito ^41^. El historiador José Toribio Polo es claro en señalar que, en 1889, terminó en Arequipa (Perú) una epidemia de sarampión ^53^. Al parecer, los contactos comerciales y el auge del cultivo del café están ligados con la rápida diseminación del sarampión. El médico Juan B. Londoño informó que, en 1885, había ocurrido una epidemia de sarampión en Salamina (Caldas), en un artículo publicado en los *Anales de la Academia de Medicina de Medellín* en 1890, luego de reportar una fuerte epidemia que ocasionó la infección de 1.800 individuos.

Su análisis permite señalar que los primeros casos ocurrieron entre menores de edad que habían estado en Pereira, donde se había presentado la epidemia. Esta epidemia llamó la atención por el gran número de muertes, que incluyó principalmente menores de 14 años (87 casos), cuya causa de muerte fue bronconeumonía; también, afectó a 7 mujeres y de 12 a 14 hombres, principalmente por complicaciones neurológicas; al parecer, la coexistencia de malaria se asoció con la mortalidad ^40^. Infortunadamente, la estimación de la incidencia y la letalidad no es posible debido a la ausencia de datos demográficos; solo se tienen datos de los censos de 1870 y 1905, un periodo muy extenso en el que ocurrieron muchos hechos, incluyendo epidemias de mortalidad variable, lo que impide dimensionar la disminución poblacional asociada con el sarampión. Solo como referencia, en 1905, se contaron en Salamina 14.140 habitantes ^54^, y debe tenerse en cuenta que el municipio experimentaba un gran flujo migratorio por ser lugar de paso ^55^.

Con gran similitud con este informe, el médico Manuel Uribe Ángel describió que esta epidemia afectó diversos municipios como Pasto, Popayán, Cali, Cartago, Pereira, Manizales, Salamina, Envigado, Pácora y Abejorral; este autor se enfocó en asuntos clínicos, como la presentación conjunta de dengue, sin describir la magnitud del evento; el médico señalaba que no tuvo tiempo de hacer autopsias debido a la carga laboral atendiendo los afectados por la epidemia ^41^. Sin embargo, este informe es muy interesante al dejar ver cómo el pensamiento "contagionista" y el "anticontagionista" permitían la comprensión de las epidemias ^56^; ya se observaba una transición en el conocimiento sobre el origen de las enfermedades.

En varios municipios de Antioquia, Caldas y Risaralda, se presentaron epidemias de sarampión en la segunda mitad del siglo XIX. Se reportaron epidemias en Titiribí (Antioquia) (1869), Salamina (Caldas) (1885), y Pereira (Risaralda) entre 1885 y 1890. Precisamente, en 1890, la epidemia se expandió a varios municipios de Antioquia y afectó nuevamente a Salamina. Entre enero y mayo de 1897, ocurrió una gran epidemia de sarampión en Bogotá, que ya se había presentado en Antioquia, Boyacá, Santander, Tolima y la costa Atlántica. La epidemia tuvo lugar concomitantemente con otras epidemias de tos ferina y fiebre tifoidea, por lo que algunos cuadros clínicos no fueron típicos. En total, hubo 620 fallecidos, cerca del 42,91 % de toda la mortalidad en ese periodo, siendo casi todos menores de edad pobres que vivían en los barrios periféricos de la ciudad ^42^.

Otra epidemia en Bogotá se inició en octubre de 1905 y se prolongó hasta junio de 1906, comenzando en el barrio de la estación del ferrocarril de la Sabana, y extendiéndose rápidamente a toda la ciudad y sus alrededores. Afectó más a los pobres en su "variedad disentérica", y a los más pudientes en la "variedad neumónica", ocasionando la muerte de 326 individuos, principalmente menores de tres años, residentes en los barrios Santa Bárbara, Las Cruces y Las Nieves ^43^^-^^45^. En este caso, es posible estimar la incidencia en 3,26 casos por 1.000 habitantes, aunque debe tenerse precaución dado que el censo de 1905 indicaba exactamente una cifra de 100.000 habitantes ^55^, lo que obviamente genera dudas. En Cartagena, Mompox y Calamar, se sabe de una epidemia de sarampión en 1915, pero no el número de casos. Sin embargo, es posible ver las medidas tomadas para su control, que incluían: la solicitud de colocar una bandera amarilla en la ventana o puerta donde había afectados, prohibir la velación de cadáveres cuya causa de muerte fuese sarampión, buscar la atención médica y destinar cien pesos para suministrar medicinas a los pobres ^57^.

En 1922, en Titiribí (Antioquia), el médico oficial Jaime Orozco informó que el sarampión estaba "haciendo su agosto", una forma de señalar su gran tamaño ^39^. En esta época, el municipio era muy importante por la minería del oro en la región. Otra epidemia importante de sarampión ocurrió en 1923 entre los siona, grupo tukano del Putumayo colombiano, la cual provocó una disminución importante de su población ^46^. Los estudios etnohistóricos han permitido determinar que era claro para los siona que el contacto con personas no indígenas interesadas en la extracción de resinas se asociaba con la epidemia, pero su cosmovisión incluía una explicación basada en batallas chamánicas entre *curacas,* curanderos poderosos de las diferentes comunidades ^46^. En medio de la guerra con Perú (1932-1934) y sus problemas sanitarios descritos por Sotomayor ^58^, una epidemia de sarampión diezmó al pueblo yagua de la frontera colombo-peruana; la muerte de una tercera parte de la población cercana a 1.000 individuos se asocia con la llegada de las tropas peruanas a la región ^47^; infortunadamente no se tiene claridad del impacto en la población colombiana.

De acuerdo con un informe del director del Departamento Nacional de Higiene, Benigno Velasco Cabrera, sabemos que

"en épocas de epidemia la mortalidad por sarampión es extraordinariamente alta, y en Bogotá en 1933 el porcentaje de mortalidad por sarampión subió a 24,2 en relación con las demás causas de defunción" ^48^.

Queda así claramente establecido el gran número de casos que podían llegar a ocasionar las epidemias de sarampión. Es importante señalar que, a comienzos del siglo XX, las epidemias eran muy relevantes en la sociedad, por lo que en su control participaban, incluso, las maestras de las escuelas. Cuando se detectaba un caso, se prohibía el ingreso a clases en los siguientes 16 días, y se brindaba educación a padres y vecinos sobre medidas de higiene ^59^. El imaginario sobre la enfermedad era tal, que incluso Jorge Eliécer Gaitán lo usó de símil para expresar que su movimiento político no era socialismo o comunismo expropiador de la riqueza: "Estamos muy lejos del sarampión extremista sin reflexión y sin método" ^60^.

Por su parte, entre los indígenas barí del norte de Venezuela, la primera epidemia de sarampión ocurrió entre 1960 y 1962. Los barí habían decidido mantenerse en aislamiento desde la expulsión de los capuchinos en 1818, y esto no cambió sino hasta 1960, cuando decidieron reestablecer relaciones pacíficas con población no indígena, lo que facilitó el incremento de la enfermedad. En 1964, la epidemia reapareció entre los barís asentados en el río de Oro y el Catatumbo, en zonas limítrofes entre Venezuela y Colombia. El impacto de estas epidemias fue catastrófico, ya que se habla de que cerca del 70 % del pueblo barí de Venezuela y Colombia falleció de sarampión entre 1960 y 1966, siendo más afectados quienes vivían en territorio colombiano ^49^^,^^50^.

De acuerdo con Walker y colaboradores ^61^, muchas otras epidemias de sarampión han impactado poblaciones indígenas amazónicas que estaban en condiciones de aislamiento. Entre todas, la epidemia de sarampión de 1968 entre los indígenas yanomami, ubicados en la frontera entre Venezuela y Brasil, merece una mención especial. Este pueblo tuvo sus primeros contactos con individuos fuera de sus comunidades hacia 1950, cuando algunos misioneros llegaron a la región. La enfermedad se introdujo por el alto Orinoco, al parecer, debido a la llegada de brasileños provenientes de la región del río Negro ^62^. De acuerdo con algunos estudios previos, la mortalidad estuvo entre el 18,7 y el 25 % de la población ^61^. En esta época, también se estaba aplicando la vacuna contra el sarampión, lo que generó una gran discusión por el posible efecto de las brigadas de vacunación como portadoras de la infección ^63^. Sin duda, un problema intercultural dificultó el entendimiento y generó molestias entre los indígenas, lo cual fue discutido ampliamente entre los antropólogos de la época ^64^. Si bien es claro que la enfermedad estaba presente en las cuencas hidrográficas del Amazonas y el Orinoco, no es clara la relación que tuvieron con las epidemias en Colombia.

La vacunación cambiaría la presentación de la enfermedad. En un pequeño estudio pionero en Colombia, realizado entre 1962 y 1964, se evaluó la eficacia de la vacuna patentada de virus inactivo de un laboratorio estadounidense, con 29 menores de edad expuestos y 12 menores como grupo control, encontrándose seroconversión en el 45 % de los vacunados ^65^. Este estudio se desarrolló en medio de las discusiones sobre la efectividad y la seguridad de las vacunas vivas y atenuadas, y empezaba a revelar el interés económico de los laboratorios privados en las vacunas, cuya investigación y producción se llevaban a cabo, tradicionalmente, en laboratorios públicos y, además, no eran patentadas ^66^. Sin duda, muestra el influjo del capital estadounidense en los médicos colombianos de la época.

**Figura 1:**
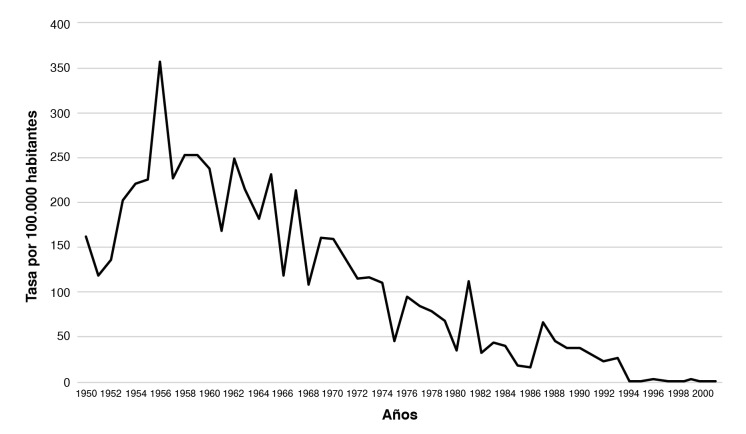
Tasa de incidencia por sarampión en Colombia por 10.000 habitantes entre 1950 y 2001; éxito de la vacunación

Pese a esto, la vacuna contra el sarampión, realmente, estuvo disponible en Colombia desde el 1984; y desde el 2000, se inició la vacunación con dos dosis para menores de cinco años y empezaron las campañas dirigidas a individuos vulnerables ^67^. El impacto de la vacunación se manifestó en el decrecimiento de los casos entre 1979 y 2018. Pese a esta disminución, la mortalidad por enfermedades prevenibles con vacunas, en ese mismo periodo, llegó a 12.201 casos, correspondientes a la mayor proporción (28,2 %) asociada con el sarampión ^68^. Si bien no hay un registro completo de todos los brotes de sarampión, el Plan Ampliado de Inmunización (PAI) permitió conocer mejor los ciclos de la enfermedad; en general, las epidemias de sarampión duraban tres años, como las de 1980 a 1982 y 1986 a 1988, con periodos interepidémicos de aproximadamente tres años. Estos tiempos sin enfermedad fueron aumentando a medida que se incrementaba la cobertura de la vacunación ^52^. Estos datos sirven para entender la dinámica epidemiológica del sarampión durante gran parte de los siglos XIX y XX, de la que no existen muchos registros. Un estudio basado en datos históricos de 1950 a 2001, permite observar la acentuada disminución del sarampión en Colombia, en gran medida, como resultado de la vacunación ^69^ (figura 1).

Datos estimados por Jiménez Peña ^67^

En 1993, el país experimentó un brote con 5.000 casos de sarampión y 48 muertes, con una tasa de incidencia cercana a 28 casos por 100.000 habitantes. En el 2002, ocurrieron 139 casos relacionados con un caso proveniente de Venezuela y luego, desde 2011 a 2015, solo se presentaron casos importados de Brasil, España, Alemania y Suiza. En los años 2016 y 2017, no hubo casos en todo el país ^51^, lo que permitió que Colombia fuera considerada libre de transmisión endémica en el 2014 y, así mismo, todo el continente americano en el 2016 ^61^. Desde mediados del 2017, los casos de sarampión procedentes de Venezuela se han convertido en una amenaza para los indígenas de Colombia, Brasil, Ecuador y Perú, como consecuencia de las escasas coberturas de vacunación y de atención, y la poca vigilancia en aquel país.

Los primeros casos de sarampión importados de Venezuela, se presentaron en el estado de Roraima, en la frontera con Brasil, afectando y afectaron los pueblos yanomami y ye'kwana, transmitiéndose entre quienes viven en regiones donde hay minería ilegal de oro ^51^. En el 2018, se presentó en Colombia un caso importado de Caracas (Venezuela) asociado con el aumento de la inmigración iniciada en el 2017. En los dos años siguientes, el Instituto Nacional de Salud registró 24 brotes, especialmente en Cartagena, La Guajira, Norte de Santander y Barranquilla, que afectaron migrantes venezolanos y casos comunitarios e intrahospitalarios relacionados ^52^. Afortunadamente, con la subsecuente vacunación en regiones de gran riesgo se logró que los brotes no superaran las 12 semanas de duración y, además, interrumpir la transmisión. Actualmente, se están incrementando los casos de sarampión en América, principalmente entre individuos no vacunados de los Estados Unidos de América ^70^, lo que ha motivado la vigilancia estrecha de la enfermedad en el país.

## Epidemia de sarampión entre los hitnü (1964)

Como se puede apreciar a partir de lo expuesto anteriormente, no hay mucha información sobre las epidemias de sarampión en territorio colombiano. Existe un gran vacío por eventos epidémicos que no se han registrado. Según la historia natural conocida de la enfermedad, esta se presenta en ciclos repetitivos de epidemias cada pocos años cuando no hay vacunación. Esto es especialmente cierto para las epidemias que ocurrieron durante los siglos XVI a XVIII y que afectaron a los indígenas. Una forma útil de mejorar la comprensión de estos hechos pasados, es mediante el estudio de epidemias más recientes que brindan información contextual de enfermedades que llegan por primera vez a un grupo poblacional. Ese es el caso del pueblo hitnü, de tradición seminómada, que habita en Arauca y había estado en aislamiento; de esta manera, la epidemia de sarampión de 1964 generó una situación similar a la que vivieron los indígenas que tuvieron contacto con europeos durante los periodos de la Conquista y la Colonia. Lo ocurrido pudo ser reconstruido recientemente con aproximaciones de etnografía histórica ^5^.

La década en la que se presentó la epidemia corresponde a un momento álgido en Arauca, particularmente en su zona selvática, donde el gobierno del presidente Guillermo León Valencia, impulsó la colonización de la selva del Sarare desde el año 1963. El presidente adjudicó créditos y capacitación a campesinos con el fin de impulsar el desarrollo agrario de la región y expandir la frontera agrícola de Colombia; este proceso se denominó "colonización dirigida" y fue continuación de la ley de la reforma agraria de 1961 ^71^^,^^72^. En el marco de esta iniciativa, se lanzó el proyecto "Arauca 1", desarrollado durante esta década. Así, se buscó que 5.000 familias víctimas de desplazamiento por violencia, provenientes de cinco departamentos de Colombia, se instalaran en 100.000 hectáreas de las selvas del Arauca, con apoyo del Instituto Colombiano de la Reforma Agraria (INCORA) y el Banco Interamericano de Desarrollo (BID) ^72^.

Esto fue fortalecido durante el periodo presidencial de Carlos Lleras Restrepo (1966-1970) ^73^, aunque para ese entonces se hablaría de una colonización "orientada", en la que colonos y gobierno participarían en la planeación de la colonización y el avance agrario ^72^. El gobierno de entonces propició el desarrollo económico de esta región, desestimando la existencia de pueblos indígenas ^74^, por lo que los nuevos colonos se apropiaron de las tierras y sus recursos. Es así como los hitnü, que tenían asignadas 18.600 hectáreas, perdieron gran parte de su territorio, hasta llegar a ser solo un tercio del área original ^75^^,^^76^. Pese a la gran extensión, debe señalarse que son terrenos inundables durante las dos temporadas lluviosas del año, las cuales disminuyen ostensiblemente las tierras habitables.

Otros elementos de interés se sumaron durante esta época, tales como el inicio de la explotación petrolera con las perforaciones del pozo La Heliera 1 y el pozo Tame, entre 1959 y 1960 ^77^. Asimismo, en 1964 surgió la guerrilla del Ejército de Liberación Nacional (ELN) ^78^ y, solo dos años después, hicieron presencia en Arauca. Sin duda, el inconformismo de los colonos con la gestión del Instituto Colombiano de la Reforma Agraria (INCORA), jugó un papel importante para la presencia guerrillera ^73^^,^^79^. Otro hecho sobresaliente en 1964 fue la alerta, por parte del gobierno municipal de Arauca, sobre la presencia de cultivos de marihuana en la zona ^80^.

En ese mismo año, Venezuela inició la construcción del puente internacional José Antonio Páez sobre el río Arauca, para comunicar a ese país con Colombia y facilitar el transporte de petróleo ^81^. Todos estos hitos, resumidos en el cuadro 2, muestran la llegada masiva de nuevos actores a las selvas de Arauca que, de alguna manera, empezaron a tener contacto con los pueblos indígenas guahibo, como los hitnü ^82^.

**Cuadro 2:** Principales hitos en la historia del pueblo hitnü (Arauca, Colombia) hasta el 2009

Periodos	Descripción
1500-1600	- Descubrimiento del territorio por alemanes y llegada de los primeros españoles a la región
1600-1700	- Llegada de las misiones jesuitas
1700-1800	- Expulsión de las misiones jesuitas
	- Llegada de los frailes capuchinos
	- Fundación de la Villa de Santa Bárbara de Arauca (antes caserío indígena arawak)
	- Fundación de cinco pueblos indígenas (guahibos) por las misiones capuchinas
1910-1920	- Creación de la comisaría especial de Arauca
1920-1930	- Incursión de los primeros colonos
1930-1940	- Los hitnü cambian de habitar río arriba a río abajo del Lipa, buscando intercambio de herramientas con los colonos.
1950-1960	- Jornadas de cacería de indios (“guahibadas”)
	- Arauca cambia de ser comisaría a ser intendencia.
	- Colonización campesina de la selva del Sarare promovida por el gobierno nacional para estimular el agro
	- Inicio de la explotación petrolera con las perforaciones del pozo La Heliera 1 y el pozo Tame
1960-1970	- Creación del partido político ANAPO (Alianza Nacional Popular); efímero auge en Arauca
	- Desplazamiento de 300 hitnü del caño Colorado hacia el hato del colono ganadero Gilberto Fernández
	- Epidemia de sarampión entre los hitnü
	- Reporte de primeros cultivos de marihuana
	- Materialización de la ley de la reforma agraria iniciada con la colonización campesina
	- Aparición de la guerrilla del Ejército de Liberación Nacional (ELN) en la región
	- Construcción del puente internacional José Antonio Páez entre Venezuela y Colombia

La epidemia de sarampión de 1964 entre los hitnü, está registrada en otros documentos ^82^^-^^84^, con muy baja difusión. Entre todos, sobresalen los registros etnográficos de los años 1978 y 1981-1982 del antropólogo Miguel Lobo-Guerrero, cuya información sobre la epidemia se publicó de manera parcial. Allí se señaló que aproximadamente 300 indígenas hitnü se desplazaron de la selva atraídos por hachas, cuchillos y telas dadas por los colonos a los chiricoas, otro grupo indígena de la zona ^82^. En 1962, se asentaron en el terreno cedido por Gilberto Fernández, un colono ganadero, para que estuvieran protegidos de los actores armados y adquirieran las costumbres de los blancos, incluyendo el vestuario para ocultar su desnudez y facilitar la consecución de alimentos. Sin duda, el cambio súbito de pasar de la vida seminómada a asentarse en un espacio reducido favoreció que, al emerger el sarampión entre los indígenas, se transmitiese rápidamente entre todos los miembros del pueblo hitnü.

Este primer contacto con el virus, en una comunidad sin vacunación, ocasionó la muerte de cerca de 100 indígenas, en su mayoría menores de edad y adultos mayores. De esta manera, la población disminuyó en una tercera parte en apenas pocos días. Esto debió ser similar, tanto en el contexto como en el impacto demográfico, con las epidemias ocurridas en diversas regiones del país durante los periodos de la Conquista y la Colonia. La llegada masiva de colonos y actores violentos al territorio, muchos en búsqueda de riquezas naturales, ocasionó el desplazamiento de los indígenas. Este es un hecho repetido en la historia y que, en el caso de los hitnü, se vio facilitado por la extracción petrolera que trajo consigo nuevas rutas de acceso a un territorio que se consideraba inhóspito. Resulta interesante que, si bien es evidente el contacto con personas no indígenas, la presentación de la epidemia se explica por las luchas entre líderes de sus comunidades, igual a como se ha relatado respecto a otros grupos indígenas ^5^.

## Discusión

Este trabajo brinda una panorámica general de las epidemias de sarampión que han ocurrido en territorio colombiano, dentro de un contexto global. La enfermedad llegó con los europeos, no existía previamente en América, y ocasionó una gran mortalidad que tuvo fuerte impacto demográfico durante la Conquista, de manera similar a lo sucedido con otras epidemias, como las de viruela ^15^. Este tipo de debacles demográficos se repitieron en diferentes momentos, incluso en la segunda mitad del siglo XX, cuando ocurrieron los primeros encuentros entre indígenas y no indígenas. Si bien hay registros disponibles sobre su acaecimiento, el comportamiento epidemiológico de la enfermedad sugiere que hay muchas epidemias no registradas. Sin embargo, pese a ser un conteo incompleto, permite observar el fuerte impacto demográfico negativo que tuvo entre las poblaciones sin inmunidad.

También, es evidente que son mejor reseñadas las epidemias ocurridas en ciudades importantes para el desarrollo económico, dependiendo del momento histórico, y es de menor calidad el registro de las epidemias que afectaron a los indígenas; esto puede asociarse con los impactos económicos negativos de las epidemias y con la presencia de personal médico que registre los hechos epidemiológicos. Con base en los conocimientos actuales, es probable que las epidemias de sarampión hayan permitido la muerte de menores de edad por infecciones oportunistas en los años posteriores a la epidemia, debido a la inmunosupresión de hasta dos años de duración ^85^. Hay registros de las epidemias de otras enfermedades posteriores a las de sarampión, pero es un tema que requiere estudios más detallados.

La vacunación marcó un hito en la historia de la enfermedad, por lo que la inclusión en el PAI produjo un declive del número de casos y epidemias. Fue tan exitoso, que llevó a la erradicación total de la enfermedad en menos de medio siglo. Lastimosamente, esta tendencia cambió en los últimos años por la reemergencia mundial del sarampión y la disminución de la cobertura de vacunación nacional por los problemas durante la vacunación contra el virus del papiloma humano ocurridos en Carmen de Bolívar en el 2014 ^86^, y la resistencia a la vacunación durante la pandemia de COVID-19 ^87^. Sin duda, la vacunación es una intervención poderosa de salud poblacional que, para tener gran efectividad, requiere acompañarse del abordaje apropiado de los factores sociales determinantes. Siguiendo a Geoffrey Rose ^88^, se puede hacer un símil señalando que la eficacia de una vacuna no asegura la efectividad de la vacunación. Para lograr el éxito, debe reconocerse que la vacunación puede mostrarse para algunas poblaciones, incluso, como un elemento de medicalización o de la llamada "farmaceuticalización" ^89^, que buscan explicar todo desde una aproximación biomédica. Así, durante las epidemias, puede correrse el riesgo de reducir la crisis sanitaria a un problema médico de gran frecuencia que debe ser abordado con medicamentos, oscureciendo el rol de los factores sociales determinantes en la génesis y en la respuesta frente a la epidemia.

La historia ha sido clara en mostrar que los diferentes contextos en los que ocurren las epidemias determinan quiénes son los individuos más afectados, siendo precisamente los más vulnerables, los cuales suelen tener mayor morbilidad y mortalidad, tal cual se observó durante las epidemias de sarampión y la pandemia de COVID-19 en Colombia ^90^. Las desigualdades previas a la epidemia, no abordadas desde los diferentes sectores sociales, afectarán cualquier intervención en medio de la crisis sanitaria. Se sabe que aquellas poblaciones con mayores desventajas económicas y desigualdades sociales, incluidas las étnicas, suelen presentar desconfianza frente a las instituciones y una baja adopción de medidas preventivas ^91^. Por ello, una adecuada preparación ante epidemias y pandemias debería incluir la disminución de las inequidades y el mejoramiento de la confianza en los tomadores de decisiones por parte de los ciudadanos ^92^. A esto se suma que, en los contextos de primer contacto marcados por la falta de inmunidad, la susceptibilidad individual ocasiona que sean el grupo con más afectados por una epidemia. El caso de los hitnü permite ver las complejidades que tiene un pueblo indígena en sus primeros contactos con los no indígenas, en el que hay un intercambio microbiológico y social y los más vulnerables suelen ser los que tienen que padecer más sufrimiento, enfermedad y muerte. Hechos similares se sucedieron en la historia de Colombia con diferentes grupos indígenas.

**Figura 2:**
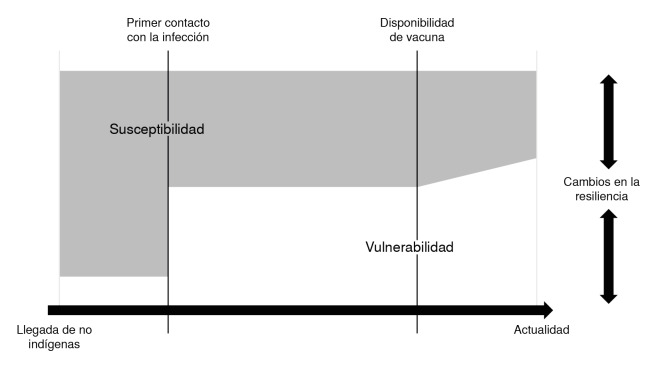
Importancia relativa de la susceptibilidad biológica y la vulnerabilidad social en la presentación de casos durante una epidemia

En conclusión, el trabajo aquí presentado permite establecer unos hitos principales que marcan la historia del sarampión en Colombia, en términos de la importancia que tienen la vulnerabilidad, la susceptibilidad y la resiliencia (figura 2), lo cual, incluso, es posible extenderlo a otras enfermedades infecciosas. Ante una enfermedad que emerge en una población, la susceptibilidad biológica juega un rol mayoritario, pero no absoluto, para determinar los afectados; la falta de inmunidad hace la diferencia en este momento. Luego, con la aparición de individuos inmunes de manera natural y los cambios inmunológicos asociados con el contacto entre diversas poblaciones, la vulnerabilidad social adopta un papel importante. Con la disponibilidad de las vacunas es posible disminuir la susceptibilidad biológica, por lo que todos los procesos asociados con la vacunación resaltan la importancia de los factores sociales determinantes; es así como la forma de distribución y el acceso a la vacuna, pueden aumentar o disminuir brechas sociales. Definitivamente, la disponibilidad de vacunas, pese a su gran importancia en la salud poblacional, no es suficiente para prevenir una enfermedad; el éxito de la vacunación depende de abordar adecuadamente los factores socioculturales determinantes.

En una vista panorámica de todas las epidemias de sarampión en Colombia, es evidente que uno de los factores sociales determinantes más importantes ha sido la violencia y sus consecuencias ^93^. Esto fue claro durante la Conquista europea y en épocas recientes; si bien hay diferencias, la historia muestra momentos en los cuales la violencia directa estuvo relacionada con la aparición de las epidemias, como sucedió durante los diferentes tipos de conflicto armado cuando hubo homicidios que llevaron a migraciones y otros cambios sociales súbitos.

En otros momentos, la violencia estructural es el contexto en el que se observan las epidemias, resultado de la situación política y económica en la que han vivido las poblaciones afectadas a lo largo de su historia ^94^, violencia que, precisamente, hace referencia a las desigualdades e inequidades que llevan a una mayor vulnerabilidad de los grupos más afectados con las epidemias. El reconocimiento de la violencia como factor determinante y del contexto de las condiciones de la salud en Colombia, pese a lo evidente que puede resultar, sigue siendo una deuda de las políticas en salud poblacional.

Este trabajo tiene limitaciones inherentes al enfoque adoptado. Al buscar una mirada panorámica de las epidemias de sarampión en Colombia, no se enfocó en detalles específicos de una epidemia, como ha sido más habitual entre historiadores, demógrafos e historiadores médicos. Sin duda, abordajes más profundos, como los de la ciudad de Segovia (España) en 1883 ^95^, servirán de ejemplo a futuros estudios de las epidemias aquí presentadas, incluyendo la búsqueda de información primaria. De igual manera, los hallazgos científicos recientes mediante técnicas de biología molecular modernas, pueden brindar evidencia paleopatológica que complemente los registros históricos.

Para finalizar, si bien las epidemias son eventos biológicos masivos, también son eventos sociales por definición. Por eso, una aproximación solo desde la biomedicina es contraria a su naturaleza y, por tanto, incompleta. El aproximarse desde un enfoque biosociocultural basado en la historia, permite una comprensión más holística y reflexiva de las reacciones frente a los eventos masivos en salud ^96^. En la actualidad, en diversos países, el sarampión reemergió entre inmigrantes no vacunados y poblaciones en condiciones de gran vulnerabilidad social ^97^, así como entre miembros de grupos antivacunas ^98^, lo que genera una amenaza mayúscula para la salud poblacional colombiana más vulnerable. Esto es más grave cuando individuos con pensamiento antivacunas llegan a cargos de poder, como en los Estados Unidos de América ^70^. Por ello, los análisis históricos resultan muy relevantes para una mejor comprensión de las crisis sanitarias generadas en las epidemias.
